# Acceptance and Commitment Therapy for Psychosis. What’s the evidence?

**DOI:** 10.1192/j.eurpsy.2022.1922

**Published:** 2022-09-01

**Authors:** F. Azevedo, R. André, L. Silva, R. Medinas, C. Almeida

**Affiliations:** 1 Centro Hospitalar de Lisboa Ocidental, Psychiatry, Lisbon, Portugal; 2 Centro Hospitalar Universitário Lisboa Norte, Psychiatry, Lisboa, Portugal; 3 Unidade Local de Saúde do Baixo Alentejo, Psychiatry, Beja, Portugal; 4 NOVA Medical School, Psychiatry And Mental Health, Lisboa, Portugal

**Keywords:** acceptance and commitment therapy, CBT, schizophrénia, therapy

## Abstract

**Introduction:**

Cognitive behavioural therapy for psychosis as an adjuvant to pharmacological treatment has been been shown to be one of the most effective interventions for schizophrenia with benefits noted in even treatment resistant schizophrenia. Benefits have been mostly registered in the positive symptoms domain of schizophrenia. Acceptance and commitment therapy is a third generation Cognitive-Behavioural Therapy, empirically supported for a range of symptoms and conditions, including psychosis, with quickly increasing data. It targets experiential avoidance, which seems to be closely related with psychopathology. Its ability to also target affective symptoms can be an important advantage in the adjuvant treatment of psychosis.

**Objectives:**

To critically review the evidence of acceptance and commitment therapy in psychosis.

**Methods:**

Non-systematic review of the literature with selection of scientific articles published in the past 10 years; by searching Pubmed and Medscape databases using the combination of MeSH descriptors. The following MeSH terms were used: “schizophrenia”, “acceptance and commitment therapy”.

**Results:**

Very few studies have been published on ACT and psychosis, with even less controlled trials and systematic reviews. So far there is convincing evidence for ACT reducing the frequency of hallucinations, increasing the outcomes of traumatic events associated with psychosis and having measurable effects on anxiety and help seeking behaviour.

**Conclusions:**

As Acceptance and Commitment therapy evolves and more evidence arises a new kind of therapy with possible effects on both affective and positive symptoms in schizophrenia can emerge, allowing us to know what works for patients with psychosis and through what mechanisms and permitting the improvement of treatment strategies.

**Disclosure:**

No significant relationships.

